# The Injunction Case of the Boston Dental College

**Published:** 1869-08

**Authors:** 


*The Injunction Case of the Boston Dental College.*—In the July No. of the *Journal* we informed our readers that an injunction had been issued against this College, at the instigation of six members of the Board of Trustees, and that a hearing was appointed for Saturday, June 19th, to see whether the injunction should be made perpetual.

A hearing was held on the day appointed, and the injunction was made perpetual. This action of the Court interferes with the issuing of diplomas to the late graduating class, and to obtain them the members of this class will be compelled to attend another full course of lectures. The charge which led to the issuing of the injunction was, that the faculty intended to graduate these gentlemen after an attendance on two courses of lectures held within one year.

